# The Landscape of Gene Expression during Hyperfilamentous Biofilm Development in Oral *Candida albicans* Isolated from a Lung Cancer Patient

**DOI:** 10.3390/ijms24010368

**Published:** 2022-12-26

**Authors:** Beata Chudzik-Rząd, Daniel Zalewski, Martyna Kasela, Rafał Sawicki, Jolanta Szymańska, Anna Bogucka-Kocka, Anna Malm

**Affiliations:** 1 Department of Pharmaceutical Microbiology, Medical University of Lublin, 1 Chodźki St., 20-093 Lublin, Poland; 2 Department of Biology and Genetics, Medical University of Lublin, 4a Chodźki St., 20-093 Lublin, Poland; 3 Department of Biochemistry and Biotechnology, Medical University of Lublin, 1 Chodźki St., 20-093 Lublin, Poland; 4 Department of Comprehensive Paediatric and Adult Dentistry, Medical University of Lublin, 6 Chodźki St., 20-093 Lublin, Poland

**Keywords:** *Candida albicans*, hyperfilamentation, biofilm, virulence, gene expression, iron metabolism, oral cavity, lung cancer, antifungal target

## Abstract

The filamentation ability of *Candida albicans* represents one of the main virulence factors allowing for host tissue penetration and biofilm formation. The aim of this paper was to study the genetic background of the hyperfilamentous biofilm development in vitro in *C. albicans* isolated from the oral cavity of a lung cancer patient. Analyzed *C. albicans* isolates (CA1, CA2, CA3) were chosen based on their different structures of mature biofilm. The CA3 isolate possessing hyperfilamentation properties and forming high biofilm was compared with CA1 and CA2 isolates exhibiting low or average biofilm-forming ability, respectively. The detailed biofilm organization was studied with the use of confocal scanning laser microscopy. The whole transcriptome analysis was conducted during three stages of biofilm development (24 h, 48 h, 72 h). In contrast to CA1 and/or CA2 isolate, the CA3 isolate was characterized by a significant upregulation of genes encoding for cell wall proteins (HWP1, PGA13, PGA44, ALS3) and candidalysin (ECE1), as well as being involved in iron metabolism (FRE1, ALS3), sulfur metabolism (HAL21), the degradation of aromatic compounds (HQD2), and membrane transport (DIP5, PHO89, TNA1). In contrast, some genes (SCW11, FGR41, RBE1) in the CA3 were downregulated. We also observed the overexpression of a few genes over time—mainly FRE1, ATX1, CSA2 involved in iron metabolism. This is the first insight into the potential function of multiple genes in the hyperfilamentous biofilm formation in *C. albicans*, primarily isolated from host tissue, which may have an important clinical impact on cancer patients. Moreover, the presented data can lay the foundation for further research on novel pathogen-specific targets for antifungal drugs.

## 1. Introduction

*Candida albicans* is a common component of human microbiota and is considered an opportunistic pathogen that is especially dangerous in immunocompromised patients [1,2]. Among superficial fungal disorders, oropharyngeal candidiasis represents a local infection of the oral mucosa that creates an important problem in modern medicine because of the high frequency of colonization of the oral cavity by *Candida* spp. (up to 70% of healthy people), as well as the prevalence of risk factors that allow for excessive yeast proliferation [3].

*C. albicans* possesses multiple virulence factors; due to the clinical implications, the major one is morphogenesis, i.e., the ability to grow in two morphological forms—yeasts and filamentous forms creating pseudohyphae or hyphae. The invasive properties of filamentous forms enable this fungus to penetrate and destroy infected tissues, as well as to escape from the host’s immune system defenses [4].

The formation of filamentous forms by yeasts was found to be connected with biofilm formation. The ability of *C. albicans* to form biofilm is dependent on many factors, i.e., slime production, cell surface properties, the presence of specific adhesins on the surface, as well as ligands present on the hosts cells [2].

It has been noticed that approximately 80% of all microbial infections are directly or indirectly connected with biofilm, which, in the case of *C. albicans*, can be defined as a highly complex three-dimensional structure of a fungal community enclosed in an extracellular polymeric substance (EPS), [2,5]. Moreover, EPS itself consists of some molecules that are crucial in pathogenesis, such as exopolysaccharides, proteins, chitins, and nucleic acid. The mature biofilm of *C. albicans* can be represented by three different morphological forms—yeast, pseudo-hyphal and true hyphae. Furthermore, biofilms of *C. albicans* can be highly diversified, so called ‘strain-dependent’, and usually range from 25 to 500 µm in thickness [6].

*C. albicans* is capable of creating biofilm in practically every part of the human body: skin, wounds, oral cavity, lungs, the bloodstream; as well as on several artificial objects, including pacemakers, tubes, caterers, implants, dentures, etc. [2]. Moreover, fungal cells that live in biofilms often exhibit unique phenotypic characteristics including high resistance to antibiotics or the ability to evade host defense mechanisms. Due to the resistance to commonly used antifungal agents, the therapy of *C. albicans* infections related to the presence of biofilm remains challenging. Additionally, the eradication of a well-developed biofilm is exceedingly difficult and requires the use of doses of antibiotics that substantially exceed the highest therapeutically attainable concentration [1]. Typically, biofilm formation consists of the initial process of cell adhesion to the surface, then the multiplication of yeast cells to create microcolonies needed for the further development of hypheal or pseudohypheal forms [7]. The time needed, as well as the final structure and composition of fungal biofilm, depend on the hydrophobicity parameter of the *Candida* cell surface, the expression level of the genes involved, the conditions of the environment, and many more, when considering the process occurring in vivo.

Many signal transduction pathways as well as transcriptional regulatory networks control the hyphal development in *C. albicans*. There are some genes that are often considered to be the main regulator factors that control biofilm formation and switch from the commensal to the pathogenic state, e.g., EFG1, BCR1, BRG1, NDT80, TEC1, and ROB1 [8]. The complexity of the biofilm production mechanism in *C. albicans* is underlined by the abundance of genes regulated by the above-mentioned regulatory factors. From a medical perspective, *Candida* isolates that are able to form robust biofilms composed of filamentous forms pose the biggest threat to patients, speeding up the infection progress and rendering antifungal therapy ineffective. At present, large efforts are being made to identify the genes involved in this process, which would not only allow for a comprehensive explanation of its molecular regulation mechanisms, but also, in the future, would provide essential information for the development of novel antifungal therapeutics [9].

The aim of our study was to analyze the genetic background of the biofilm development, along with its structural organization, in three oral *C. albicans* strains isolated from lung cancer patients, with a special emphasis on the hyperfilamentous biofilm.

## 2. Results

### 2.1. Biofilm Structure and Development Analysis by Confocal Scanning Laser Microscopy

**Figure 1:**
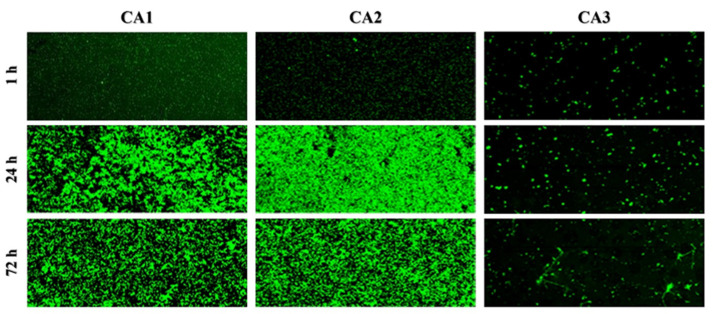
Two-dimensional scans of adhesion (1 h), early biofilm formation (24 h) and the mature biofilm (72 h) of *C. albicans* isolates (CA1, CA2, CA3) dyed with concanavalin A Alexa Fluor 488 conjugate (magnification ×50). The pictures of the CA1 and CA2 isolates are characterized by the presence of an increasing number of morphological forms in time, namely blastospores or true hyphae with sparse blastospores, respectively. The CA3 pictures at the three investigated time points are similar because the isolate proliferated only into the hypheal form, and the blastospores responsible for the “green image” in the CA1 and CA2 isolates were not present.

The biofilm structural organization of three *C. albicans* isolates (CA1, CA2, CA3) was observed with the use of confocal scanning laser microscopy (CSLM). Representative pictures of the biofilm morphology after 1 h (the process of adhesion), 24 h (early-stage biofilm formation), and 72 h (mature biofilm) are shown in Figure 1.

**Figure 2:**
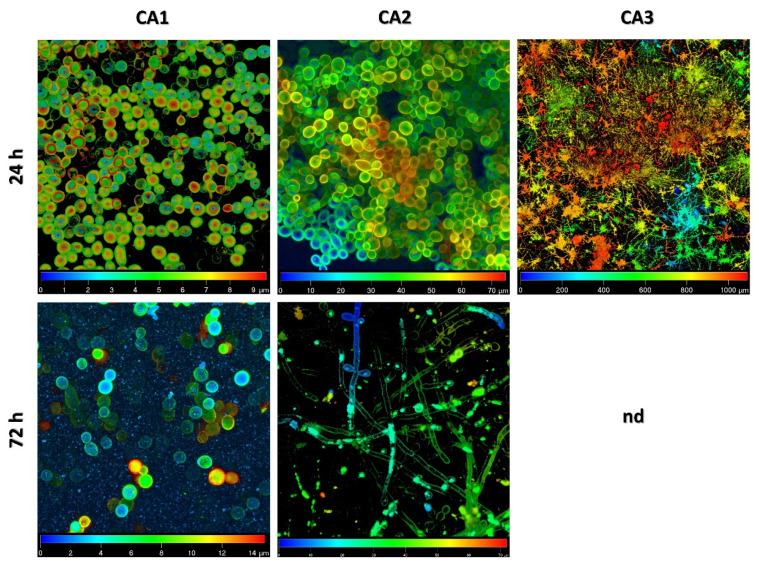
Hypsometric maps of the structure of 24 h and 72 h biofilm of *Candida albicans* isolates (CA1, CA2, CA3); nd—not determined. The CA3 isolate biofilm after 72 h was higher than the technical capacity of the confocal scanning laser microscope to produce a picture.

Based on the two-dimensional pictures, we analyzed parameters describing the steps of biofilm formation (areal porosity, length of edge line, length of skeleton line, thickness of biofilm), which was presented in detail in our previous paper [10]. A gallery of pictures presenting the three-dimensional reconstruction of 24 h and 72 h biofilms of *C. albicans* (Figure 1) showed the structure of the biofilm of each isolate (CA1, CA2, CA3) as a mixture of blastopores, pseudo-hyphal forms, or true hyphae. We observed that CA1 biofilms at 24 h and 72 h consisted of a mixture of blastopores only. The biofilm of the CA2 isolate after 24 h of incubation contained the mixture of blastopores, whereas the mature biofilm (after 72 h of incubation) comprised filamentous forms, i.e., pseudo-hyphal forms—true hyphae together with sparse blastospores included. Finally, the CA3 isolate after 24 h comprised filamentous forms—pseudo-hyphal forms and true hyphae. Moreover, in the confocal scanning laser microscopy the hyperfilamentous forms resembled "spruce forest”. The hypsometric maps illustrating the different heights of the 24 h and 72 h biofilms of CA1, CA2, CA3 isolates are shown in Figure 2. The height of the biofilm of CA1 isolate after 24 h of incubation was 7.6 µm, while the mature biofilm was 13.3 µm. The biofilm of CA2 after 24 h and after 72 h of incubation was 74.48 and 66.42 µm, respectively. The third isolate of *C. albicans* (CA3) produced the highest biofilm. The height of CA3 biofilm after 24 h was 1090 µm, whereas after 72 h it was so high that the CSLM could not produce the picture.

### 2.2. Differentially Expressed Genes between Candida albicans Isolates

**Figure 3:**
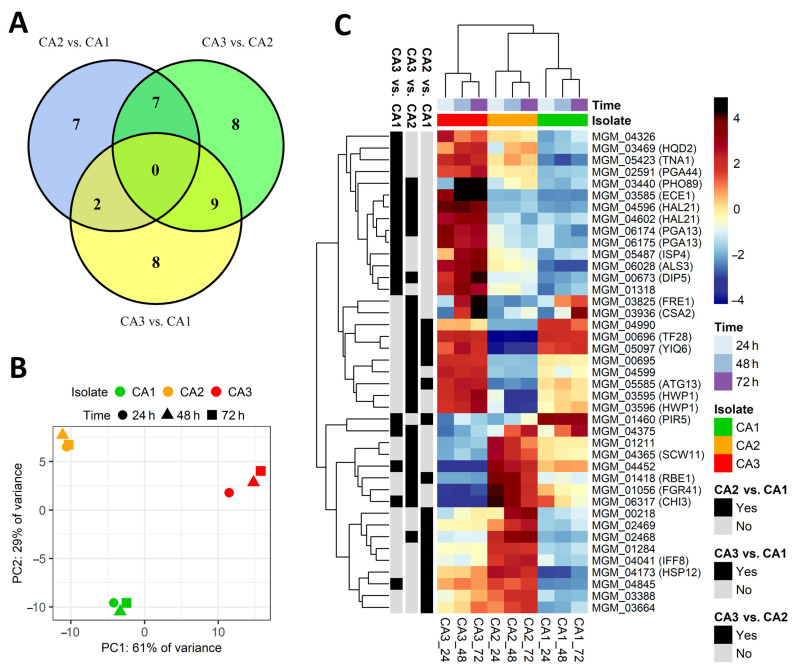
Differential expression of genes selected from CA2 vs. CA1, CA3 vs. CA1, and CA3 vs. CA2 comparisons. (**A**) Venn diagram showing intersections between groups of genes selected from the performed comparisons. The non-overlapping numbers denote the genes that were unique for each isolate, while overlapping numbers represent mutual differentially expressed genes. There were no genes common for all three comparisons. (**B**) PCA plot for the expression of selected genes. (**C**) Heatmap illustrating the expression of selected genes in the studied samples. Hierarchical clustering was performed using the average method applied to Canberra distances.

We investigated changes in the level of genes expression in three *Candida albicans* isolates (CA1, CA2 CA3) at three time points—after 24 h, 48 h and 72 h of the biofilm formation process. All three isolates exhibited large phenotypic diversity of biofilm-related structures produced in vitro. In total, a whole transcriptome analysis identified 6359 differentially expressed genes. From all of these genes, only those that met the criteria of the mean of the read counts above 100 across samples belonging to the compared groups, Benjamini-Hochberg adjusted P value below 0.05, and absolute log2(fold change) value greater than 5, were considered significantly differentially expressed. Following these criteria, 16 genes were considered dysregulated in CA2 vs. CA1, 19 genes in CA3 vs. CA1, and finally, 24 genes in the CA3 vs. CA2 comparison (Table 1, Figure 3). The obtained groups of genes were compared on a Venn diagram (Figure 3A), which shows that no genes were common for all the gene groups; however, there some genes were common for each two groups of genes. The differential character of the selected genes is illustrated in the PCA plot (Figure 3B) and the heatmap with hierarchical clustering (Figure 3C). Figure S1 presents volcano plots showing the arrangement of the negative log10 of the P values and log2 fold changes for all differentially expressed genes.

**Figure 4:**
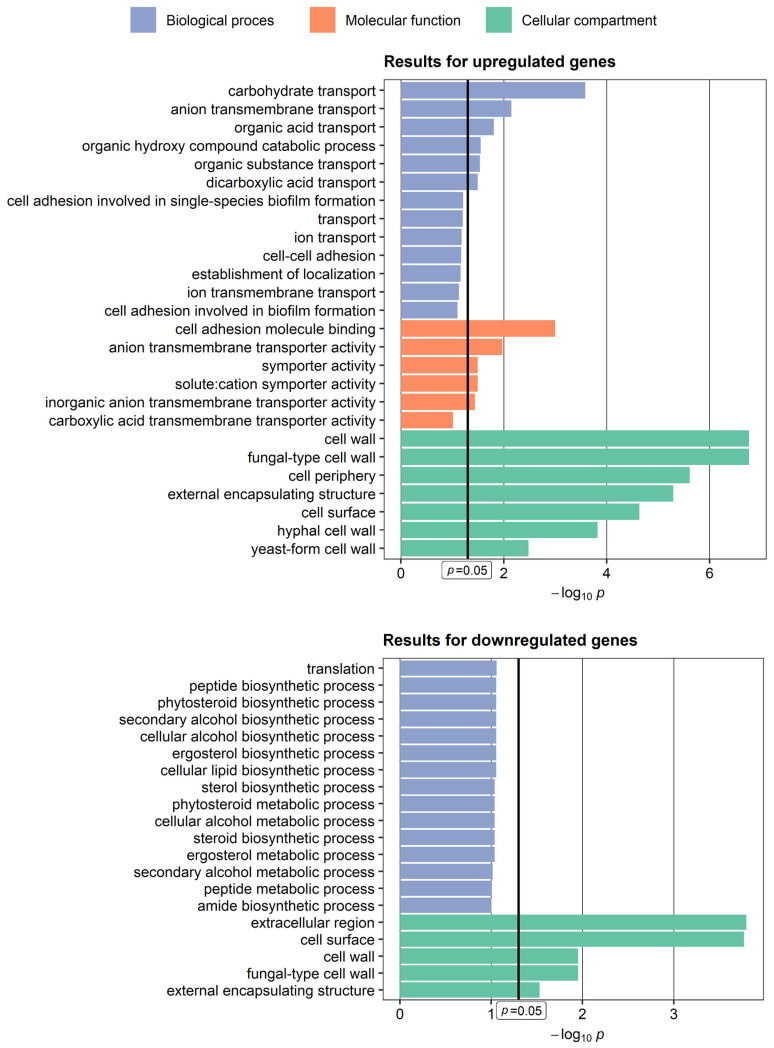
Results of Gene Ontology functional analysis performed for up- and downregulated genes selected in this study.

The GO terms that are associated with up- and downregulated groups of genes are presented on Figure 4. The majority of terms are related to transmembrane transport, cell adhesion, biofilm formation, ergosterol synthesis, and cell wall. These results clearly show that the selected genes are associated with cell morphology, showing consistency with the different microscopic images of the studied *C. albicans* isolates.

Commensurably to the level of morphological diversification, the greatest difference in gene regulation was observed in the CA3 isolate when compared to the two remaining yeast isolates. Moreover, these differences were mainly associated with genes upregulation (Table 1). First of all, the CA3 isolate was characterized by the upregulation of multiple genes responsible for the production of cell wall proteins (ALS3, HWP1, PGA13, PGA44); most of them are known to be responsible for the processes of colonization, adhesion, or the development of hyphae. Secondly, we observed a high expression of genes involved in various metabolic processes, such as FRE1, which is responsible for iron metabolism, HAL21 for sulfur metabolism, or HQD2 for the degradation of aromatic compounds. Finally, we identified several upregulated membrane transporters (DIP5, PHO89, TNA1) and a gene encoding an important virulence factor—candidalysin (ECE1). Interestingly, we observed a group of genes that were significantly downregulated in the CA3 isolate. Except for multiple genes encoding for uncharacterized proteins, we observed the presence of genes encoding for cell wall proteins (FGR1, RBE1, or SCW11) often secreted by yeast cells as important virulence factors.

As illustrated in Figure 3, the overall gene expression differences between the CA1 and CA2 isolates were mostly smaller. The isolate characterized by biofilm comprising blastospores only—CA1—possessed some significantly upregulated genes; however, the function of most of them is not yet fully understood. Among them, we found pir protein family member gene PIR5 or unknown transporters (TF28, YIQ6). A similar situation was found in the case of genes upregulated in the CA2 isolate, mainly encoding for cell wall proteins (e.g., RBE1, IFF8) or lipid-binding membrane protein (HSP12).

### 2.3. Differentially Expressed Genes between Incubation Time Points

**Figure 5:**
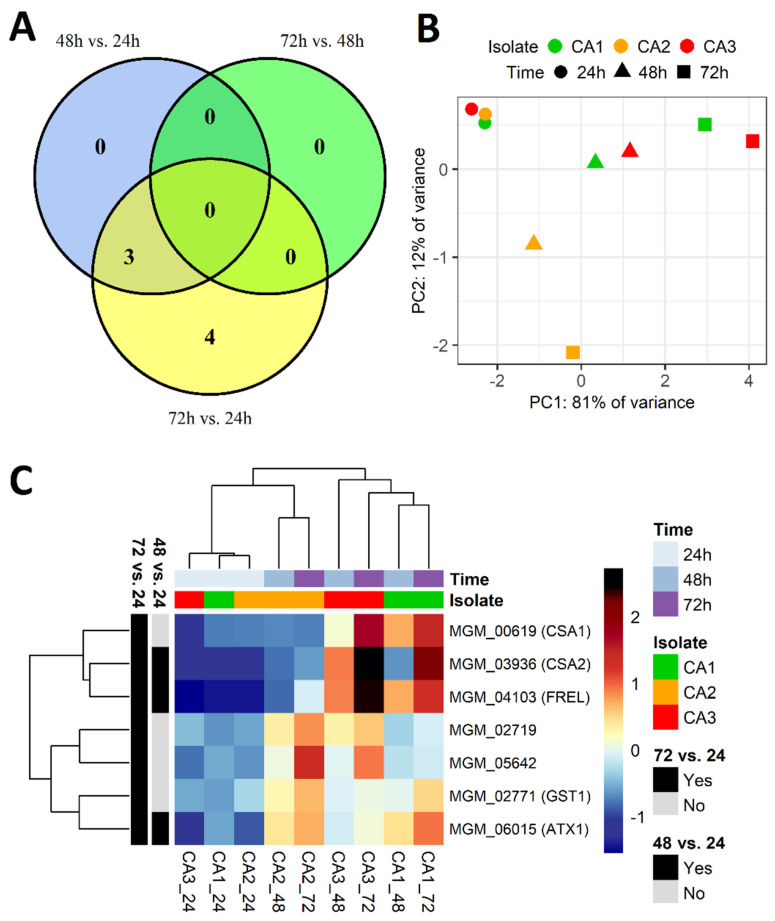
Differential expression of seven genes in *Candida albicans* isolates (CA1, CA2, CA3) selected from 48 h vs. 24 h, 72 h vs. 48 h, and 72 h vs. 24 h comparisons. (**A**) Venn diagram showing intersections between groups of genes selected from the performed comparisons. There are three genes common for the 48 h vs. 24 h and 72 h vs. 24 h comparisons, while no genes were selected from the 72 h vs. 48 h comparison. (**B**) PCA plot for the expression of seven selected genes. (**C**) Heatmap illustrating the expression of selected genes in the studied samples. Hierarchical clustering was performed using the average method applied to Canberra distances.

The differences in gene expression level could be clearly observed between particular *C. albicans* strains; however, it appeared much more stable at the three investigated time points (24 h, 48 h, 72 h) of the biofilm formation process. Genes with a different expression in time were rare (Figure S2); therefore, we lowered the log2(fold change) selection criterion from 5 to 2 (*p*-value and mean of read counts criteria were unchanged), giving seven upregulated genes (Figure 5). Three genes were selected from the 48 h vs. 24 h comparison, four genes from the 72 h vs. 24 h comparison, while no genes met the selection criteria from the 72 h vs. 48 h comparison. Among the selected genes, there were genes involved in the metal metabolism (FRE1, CSA1, ATX1, CSA2), including a CSA1 gene encoding surface antigen responsible for the elongation of hyphae, and a GST1 gene encoding glutathione S-transferase of unknown function (Table 2). The observation that significant differences in gene expression occur between 24 h and later time points (48 h and 72 h) may suggest that the main changes in transcriptome occurred during the initial steps of biofilm formation.

## 3. Discussion

Biofilm formation constitutes one of the most important virulence factors in *C. albicans*. Starting from the complex relationship with the host immune system and other microorganisms, through strain-dependent biofilm-forming abilities, and finally, preventing successful eradication from the infection site, this structure remains a challenging object of interest in fungal pathogenesis [11,12]. Despite many efforts to understand the mechanisms of biofilm formation, the process is still not fully explained on a molecular level. In our studies, we investigated the biofilm formation process in three strains of *C. albicans* (CA1, CA2, CA3), including microscopic imaging as well as whole-transcriptome sequencing to gain a detailed insight into this process. Three *C. albicans* strains were chosen on the basis of extremely different biofilm-forming abilities. After 72 h of incubation, the CA1 biofilm consisted of blastospores only, while the CA2 of blastospores and filamentous forms reached 13.3 and 66.4 µm, respectively. The third isolate was characterized by hyperfilamentation and unusual biofilm reaching over 1 mm just after 24 h (Figure 2).

It is worth pointing out that the colonization of the oral mucosa by hyperfilamentous *C. albicans* strains with strong potential to invade epithelium may be regarded as a high-risk factor, not only for oropharyngeal candidiasis, but also for systemic fungal infections. Moreover, the filamentation/hyperfilamentation process and its genetic background in *C. albicans* represents an important and as yet under-explored target for novel antifungals [13]. Invasive fungal infections, including those caused by *Candida* spp., remain a challenging therapeutic issue in cancer patients. The application of antifungals in both empirical and pre-emptive therapy is still connected with multiple side effects in patients, as well as the risk of acquiring antifungal resistance in fungi. The development of new antifungals, especially for pathogen-specific targets, would significantly improve the therapy of invasive fungal infections in immunocompromised patients, leading to a decrease in both mortality and morbidity rates [14,15,16]. The need for novel antifungals is underlined by the fact that the functional analysis conducted in our study showed the downregulation of genes involved in ergosterol biosynthesis. Ergosterol is not only the most prevalent sterol in fungi, but most of all, the main target for commonly used antifungal drugs such as amphotericin B and azoles. Mukherjee et al. noticed that *C. albicans* cells present in biofilm exhibit higher tolerance towards antifungals than cells in planktonic cultures, which raises concerns about the efficiency of routine antifungal therapy for invasive infections connected with biofilm formation [17,18].

### 3.1. Genes Upregulated in Candida albicans Isolate Exhibiting Hyperfilamentation

We observed that the high-biofilm forming ability in CA3 was connected with the upregulation of multiple genes encoding for cell wall proteins (HWP1, PGA13, PGA44, ALS3) and candidalysin (ECE1), as well as involved in iron metabolism (FRE1, ALS3), sulfur metabolism (HAL21), the degradation of aromatic compounds (HQD2), and mem-brane transport (DIP5, PHO89, TNA1).

ALS3 is a well-known surface protein classified as an adhesion molecule that enables fungal cells to attach to the colonized surface [19]. Our studies showed a significant overexpression of the ALS3 gene in CA3 exhibiting high biofilm in contrast to its low expression in CA1, characterized by a low biofilm-forming ability. The low expression of ALS3 would explain the absence of filamentous morphogenesis in the CA1 isolate, as adhesin mutants have already been reported to be defective in biofilm formation [20]. Additionally, some authors have also proven that the biofilm-forming capability in *C. albicans* is often connected with high expression of the ALS3 gene [21].

Similar to ALS3, a high expression of ECE1 is undoubtedly involved in the formation of a complex biofilm. Nevertheless, studies have also shown that the deletion of this gene has no adverse effect on mature biofilm formation [22]. Moreover, ECE1 is a crucial gene responsible for enhanced virulence because it encodes candidalysin, a cytolytic peptide that plays an important role in mucosal infections [23]. *Candida albicans* strains with a high expression of ECE1 might pose a threat in infected individuals, driving fungal pathogenicity in a multidirectional way. Furthermore, some studies present evidence that candidalysin, due to its strong inflammatory properties, is responsible for the malignant transformation of mucosal cells during infection [24]. Another strongly upregulated gene in CA3 is HWP1, which plays an important role in the development of hyphae. Additionally, it allows fungal strains to bind to epithelial cells and start the colonization process [25]. Both HWP1 and ALS3 encode proteins that are required to start biofilm formation in vivo; however, based on their characteristics, it is suggested that they have distinct functions in this process. Nobile et al. tested the hypothesis that HWP1 and ALS3 act as complementary surface proteins in *C. albicans* by mixing two mutants deprived of one of these two genes, resulting in a biofilm-competent mixture of fungal cells. Moreover, the applied models confirmed this relationship both in vivo and in vitro [26].

The upregulation of genes not directly connected with the adhesion or filamentation process in complex-biofilm formers has also been observed by other authors. Similar to our studies, a high expression of DIP5 gene encoding for dicarboxylic amino acid transporter was observed in yeasts forming complex mature biofilm, which additionally, was involved in the so-called invasive phenotype [27,28]. Another gene significantly upregulated in the CA3 isolate—HAL21, is involved in sulfur assimilation. So far, no clear connection has been established between increased HAL21 expression and biofilm formation; however, an interesting hypothesis has been presented. Studies conducted by Murillo et al. showed the upregulation of the entire sulfur assimilation pathway in the early stage of biofilm formation in *C. albicans* [29]. The authors explained this observation by suggesting the existence of a futile sulfur cycle in biofilm cells responsible for the production of glutathione (GSH) as a protection agent against reactive oxygen and the storage of cysteine.

The prevalence of upregulated genes in the *C. albicans* strain producing robust biofilm emphasizes the significance of a vast network of genes regulating this process.

### 3.2. Genes Downregulated in Candida albicans Isolate Exhibiting Hyperfilamentation

Most of the differentially expressed genes in the studied *C. albicans* clinical isolates were genes upregulated in the CA3 isolate when compared to the remaining two, thus, supporting the filamentation process. However, in the same isolate, we also observed the presence of genes with extremely low expression (FGR41, RBE1, SCW11).

Studies have shown that FGR41, among many other genes, constitutes a specific co-expression regulatory network involved in the development of candidiasis. Moreover, during the transition to true hyphae, FGR41 is often downregulated, as was the case for the CA3 strain characterized by a high biofilm formation ability [27]. Other authors found a similar situation as that in our studies that, in the case of the RBE1 gene that belongs to the putative novel family of Pry-proteins and located in the fungal cell wall, is secreted by yeast cells, playing a significant role as a virulence factor [30]. Some authors noticed that RBE1 could be downregulated under hyphae-inducing conditions, which would explain its low expression level in CA3 that creates biofilm comprising complex filamentous forms. What is also worth noticing is that RBE1 is regulated by other genes involved in the process of biofilm formation, i.e., the transcription factor EFG1 [31,32].

Another gene found with low expression in the CA3 isolate is SCW11 (Figure 3C), which encodes a putative *C. albicans* glucanase; its expression depends on many conditions [33]. Some authors noticed that its expression can be lower in hyphae than in yeast cells [34], which is supported by our observations. Namely, SCW11 expression was significantly lower in CA3 compared to CA1, whose biofilm consisted of blastospores only, and compared to CA2, producing a mixture of blastospores and filaments.

We also observed the downregulation of one of four known chitinase genes in *C. albicans*—CHI3. This gene encodes for chitinase, which is necessary for fungal blastospores to completely separate after the cell division process; this has already been investigated in *C. albicans* mutants deprived of various chitinase genes [35]. It was also observed that yeast cells often increase the content of chitin in their cell wall in stress conditions [36].

### 3.3. Iron Metabolism in Candida albicans Is Involved in the Process of Hypheal Morphogenesis

Iron constitutes an essential micronutrient in fungal pathogenesis, which is the reason for the expanded acquisition mechanisms and metabolic pathways present in *C. albicans* [37]. On the one hand, during fungal infection, the iron demand is higher at an early stage of infection when the tissue physiological state remains more or less unchanged, rather than at an advanced stage, when the destroyed tissues release iron [38]. On the other hand, in the case of the CA3 isolate characterized by hyperfilamentation, we not only observed a strong upregulation of genes involved in iron metabolism (ALS3, FRE1), but also a significant increase in expression over time in the case of FRE1, ATX1, and CSA2.

CSA2 belongs to the Common in Fungal Extracellular Membranes (CFEM) protein superfamily, and until recently, its only known molecular function was heme-iron acquisition; however, recent investigations have revealed that it could be essential for normal biofilm formation—in particular, in the yeast-to-hyphae morphological switch [39]. Our analysis showed that it was significantly upregulated in the CA3 isolate and, in addition, its highest expression was observed after 72 h, which could prove that it is also necessary for *C. albicans* to exhibit a high biofilm. The role of the other gene in biofilm formation—FRE1—also known as CLF1, encoding ferric reductase, has not been reported so far. Except for the upregulation of genes directly involved in iron metabolism, we observed the upregulation of other genes, e.g., ATX1, encoding cytosolic copper metallochaperone probably involved in the regulation of iron ion transmembrane transport, based on the function of orthologous genes in yeasts. The gene described earlier—ALS3—is also responsible for reductive iron assimilation as a ferritin receptor [40]. Despite the fact that the upregulation of genes involved in iron metabolism is clearly connected with yeast-to-hyphae transition in *Candida*, there is evidence that this regulatory network has a strong restoration capacity; thus, the absence of certain genes does not necessarily lead to the impartment of biofilm-forming abilities [41].

### 3.4. Limitations of the Study

Despite novel findings concerning the gene expression landscape in hyperfilamentous *C. albicans*, our study has some limitations. Based on the results obtained for a limited number of *C. albicans* isolates, it is difficult to extrapolate our observations to all fungal isolates exhibiting a high biofilm-forming ability, and to distinguish genes crucial for the hyperfilamentation process. Thus, further studies on this subject should investigate a wider range of *C. albicans* isolates with this feature for changes in the expression of newly described genes potentially connected with biofilm formation. Furthermore, determining whether these genes are necessary for fungal strains to exhibit hyperfilamentation would facilitate marking novel molecular pathways as potential targets for antifungal drugs.

## 4. Materials and Methods

### 4.1. Candida albicans Strains and Culture Conditions

In this study, three clinical strains of *Candida albicans* (CA1, CA2, CA3) were isolated from the oral cavity of patients who underwent lung cancer pulmonary resection. After isolation and identification, the fungal strains were stored at −70 °C in Sabouraud Glucose Broth (SB; Oxoid, Basingstoke, UK) containing 50% glycerol. Before all experiments, the strains were cultured on Sabouraud Dextrose Agar (SA; Oxoid, Basingstoke, UK) at 30 °C for 48 h, and then subcultured on SB and incubated at 30 °C for 48 h.

### 4.2. Confocal Scanning Laser Microscopy Analysis

The standardized suspension of each isolate of *C. albicans* (optical density of 0.5 McFarland standard, i.e., 5 × 10^6^ colony-forming units in mL) was prepared in SB. The following analysis involved examining three stages: the adhesion process (after 1 h of incubation), early-stage biofilm formation (after 24 h of incubation), and mature biofilm formation (after 72 h of incubation). To determine the adhesion process, 350 µL of inoculum was added to four out of eight wells of a polystyrene culture chamber and then incubated for 1 h at 35 °C. After that, the wells were gently washed with sterile phosphate-buffered saline (PBS) to remove nonadherent cells. Subsequently, 200 µL of solution containing 0.1 mg/mL of concanavalin A Alexa Fluor 488 conjugate (CAAF) was added to each well and incubated for 45 min at 35 °C. To determine the biofilm formation at an early stage, 350 µL of inoculum was added to four wells and incubated for 24 h at 35 °C. Next, the wells were gently washed with PBS to remove nonadherent cells, and 200 µL of solution containing CAAF was added to each well. The mixture was incubated for 45 min at 35 °C. To determine the mature biofilm formation, 350 µL of inoculum was added to four out of the eight wells of the culture chamber and then incubated for 72 h at 35 °C. After that, the wells were gently washed with PBS to remove nonadherent cells. Approximately 200 µL of solution containing CAAF was added to each well; then, the mixture was incubated for 45 min at 35 °C. All assays were conducted in four replicates. The pictures for the planimetric measurements were carried out in two-dimensional scans at a magnification of x 50. The planimetric analysis was performed using Image J. v. l. 36b, Wayne Rasband, National Institutes of Health, USA.

### 4.3. Total RNA Preparation

Fungal biofilm recovered from a single well of a six-well plate was centrifuged and resuspended in 800 μL of Fenzol (A&A Biotechnology, Gdańsk, Poland). The cells were placed in a tube containing 0.8 mL of zirconia beads (0.1 mm in diameter) and disrupted in a bead-beater (FastPrep24 instrument, MP Biomedicals, Santa Ana, CA, USA MP Biomaterials) at the highest power by two 30 s pulses, separated by 5 min of chilling on ice. All other steps were performed with the Total RNA Mini Kit (A&A Biotechnology, Gdańsk, Poland) according to the manufacturer’s instructions. Genomic DNA was eliminated with deoxyribonuclease I (Sigma Aldrich, Dorset, UK). The DNA removal was accomplished by adding 10 μL of 10 x reaction buffer and 10 μL of DNase I (1 unit/μL) to an 80 μL RNA sample. After 15 min of incubation at room temperature, the reaction was stopped by the addition of 10 μL of Stop Solution. Next, the sample was heated at 70 °C for 10 min to denature both the DNase I and the RNA. The RNA purity and concentration were measured spectrophotometrically, aliquoted, and stored at –80 °C for further use.

### 4.4. RNA Sequencing

RNA sequencing was conducted on three *C. albicans* isolates (CA1, CA2, CA3) at three time points during biofilm formation (24 h, 48 h, 72 h). The removal of ribosomal RNA and the isolation of poly(A) RNA from the total RNA samples were performed using the NEBNext Poly(A) mRNA Magnetic Isolation Module (New England Biolabs, Ipswich, MA, USA), according to the manufacturer’s protocol. Libraries for sequencing were prepared using the NEBNext Ultra II Directional RNA Library Prep Kit (New England Biolabs, Ipswich, MA, USA), according to the manufacturer’s manual. The quality control of the isolated RNA and prepared libraries was performed using an Agilent 2100 Bioanalyzer with an Agilent RNA 6000 Pico Kit and Agilent High Sensitivity DNA Kit (all Agilent Technologies, Santa Clara, CA, USA), respectively. Sequencing was carried out using the HiSeq4000 platform (300 cycles mode) and the reagents included in the HiSeq 3000/4000 SBS Kit (both Illumina, San Diego, CA, USA). The experiments were performed in duplicate, giving a total of 18 sequenced samples (three isolates at three time points in duplicates). The average number of raw reads obtained in the studied samples was 21,569,234 (Figure S3).

### 4.5. Sequencing Data Analysis

Adapters and reads shorter than 15 bp were removed with the use of Cutadapt in paired-end mode [42]. The sequences were aligned to 6,388 genes in the reference genome of *C. albicans* P75063 (GCA_000775525) using TopHat [43] in fr-forststrand and no-novel-juncs mode. After alignment, the number of reads for each gene was counted with the use of HTseq [44]. The Basic Local Alignment Search Tool (BLAST, https://blast.ncbi.nlm.nih.gov/Blast.cgi, accessed 24 October 2022) was used to search the UniProt database (https://www.uniprot.org/, accessed 24 October 2022) to find genes homologous to the analyzed transcripts.

Differences in expression profiles between the three *C. albicans* isolates and three time points were analyzed using the DESeq2 1.36.0 package [45] in R version 4.2.0 [46]. Prior to the analysis, the read counts of the technical replicates were merged using the collapseReplicates() function, genes with means of read counts lower than 1 were filtered out, and the final data containing 6359 gene identifiers (in the case of isolates comparison) and 6282 gene identifiers (in the case of time points comparisons) were normalized using the DESeq() function. The uniformity of the normalized expression data was confirmed on the boxplot of Cook’s distances (Figure S4). The detailed expression data of all differentially expressed genes analyzed in each sample using isolates or times of incubation as a condition are shown in Figures S5 and S6.

A graphical representation of differently expressed genes between *C. albicans* isolates was produced on volcano plots and Principal Component Analysis (PCA) plots generated using the ggplot2 3.3.6 package in R (https://cran.r-project.org/web/packages/ggplot2/index.html, accessed 24 October 2022), as well as on heatmaps with hierarchical clustering created using the pheatmap 1.0.12 package in R (https://cran.r-project.org/web/packages/pheatmap/index.html, accessed 24 October 2022). A Venn diagram presenting intersections between groups of genes selected from the performed comparisons was created using the VennDiagram 1.7.3 package in R (https://cran.r-project.org/web/packages/VennDiagram/index.html, accessed 24 October 2022).

The biological role of the selected genes was explored by a functional overrepresentation analysis, using the GO Term Finder tool from the Candida Genome Database [47]. The analysis was performed for GO (Gene Ontology) terms of *Candida albicans* and separately for up- and downregulated genes.

## 5. Conclusions

A transcriptional regulatory network controlling the hyperfilamentous biofilm formation by oral *C. albicans* strain CA3, isolated from lung cancer patients, involved the dysregulation of multiple genes, including those responsible for the processes of colonization, adhesion, and yeast-hyphae transition, as well as those involved in iron or sulfur metabolism. Knowledge about the genetic background of the hyperfilamentation process in *C. albicans* strains isolated primarily from the host organism may be useful for the development of novel pathogen-specific targets for antifungals with a high potential to prevent and/or eradicate overgrown biofilms.

## Figures and Tables

**Table 1:** Differential expression characteristics of genes in *Candida albicans* isolates (CA1, CA2, CA3).

Gene ID ^1^	Gene Name ^2^	Description ^3^	Mean Expression ^4^	Log2(Fold Change)	*p*
***CA2*** **vs. *CA1 upregulated genes***					
MGM_04173	HSP12	Heat shock protein	7359.3	6.38	2.47 × 10^-76^
MGM_02468		Uncharacterized	1306.1	6.44	1.54 × 10^-68^
MGM_01284		Uncharacterized	123.7	5.83	3.42 × 10^-35^
MGM_01418	RBE1	Pry family cell wall protein	1370.4	5.03	2.14 × 10^-30^
MGM_04041	IFF8	Putative GPI-anchored adhesin-like protein	141.5	5.50	5.07 × 10^-23^
MGM_03388		Uncharacterized	171.4	5.39	5.02 × 10^-19^
MGM_02469		Uncharacterized	110.6	6.10	5.46 × 10^-18^
MGM_04845		Uncharacterized	182.6	9.96	1.25 × 10^-14^
MGM_03664		Uncharacterized	150.3	5.72	2.21 × 10^-14^
MGM_00218		Uncharacterized	220.2	5.65	8 × 10^-7^
***CA2* vs. *CA1 downregulated genes***					
MGM_00696	TF28	Transposon Tf2-8 polyprotein	6275.0	−8.75	1.73 × 10^-219^
MGM_05097	YIQ6	Uncharacterized transporter YIL166C	4152.0	−7.12	5.38 × 10^-62^
MGM_04990		Uncharacterized	134.6	−10.65	6.99 × 10^-16^
MGM_05585	ATG13	Autophagy-related protein 13	295.8	−6.41	1.01 × 10^-14^
MGM_01460	PIR5	Cell wall Pir protein	552.2	−5.55	3.1 × 10^-9^
MGM_00695		Uncharacterized	110.6	−7.63	7.41 × 10^-9^
***CA3* vs. *CA1 upregulated genes***					
MGM_05423	TNA1	Putative nicotinic acid transporter	1693.7	6.34	8.64 × 10^-83^
MGM_06028	ALS3	Cell wall adhesin	1232.6	8.50	2.75 × 10^-81^
MGM_04596	HAL21	3’-phosphoadenosine 5’-phosphosulfate phosphatase	542.9	9.46	7.5 × 10^-53^
MGM_01318		Uncharacterized	9938.0	5.99	1.55 × 10^-51^
MGM_04602	HAL21	3’-phosphoadenosine 5’-phosphosulfate phosphatase	391.3	6.04	2.75 × 10^-48^
MGM_00673	DIP5	Dicarboxylic amino acid permease	13,615.4	6.68	3.33 × 10^-43^
MGM_06175	PGA13	GPI-anchored cell wall protein	11,790.7	5.44	1.02 × 10^-41^
MGM_03585	ECE1	Candidalysin	910.7	10.70	1.31 × 10^-40^
MGM_06174	PGA13	GPI-anchored cell wall protein	11,682.1	5.84	3.27 × 10^-36^
MGM_02591	PGA44	Putative GPI-anchored protein	200.6	5.71	8.22 × 10^-32^
MGM_04326		Uncharacterized	186.4	5.23	2.78 × 10^-16^
MGM_03469	HQD2	Catechol 1,2-dioxygenase	256.3	5.26	1.46 × 10^-15^
MGM_04845		Uncharacterized	182.6	9.12	1.62 × 10^-12^
MGM_05487	ISP4	Sexual differentiation process protein isp4	490.5	5.82	6.45 × 10^-10^
MGM_03440	PHO89	Putative phosphate permease	1088.7	7.31	4.2 × 10^-7^
***CA3*** **vs. *CA1 downregulated genes***					
MGM_04452		Uncharacterized	15,450.6	−5.13	2.03 × 10^-57^
MGM_01460	PIR5	Cell wall Pir protein	552.2	−6.24	2.17 × 10^-11^
MGM_06317	CHI3	Chitinase	11,032.2	−5.42	6.29 × 10^-11^
MGM_04375		Uncharacterized	280.6	−5.76	9.21 × 10^-4^
***CA3* vs. *CA2 upregulated genes***					
MGM_00696	TF28	Transposon Tf2-8 polyprotein	6275.0	9.10	1.33 × 10^-237^
MGM_04596	HAL21	3’(2’),5’-bisphosphate nucleotidase 1	542.9	7.72	8.31 × 10^-81^
MGM_05097	YIQ6	Uncharacterized transporter YIL166C	4152.0	7.57	2.82 × 10^-70^
MGM_03585	ECE1	Candidalysin	910.7	8.35	1.72 × 10^-54^
MGM_04599		Uncharacterized	272.0	5.92	6.32 × 10^-53^
MGM_04602	HAL21	3’-phosphoadenosine 5’-phosphosulfate phosphatase	391.3	6.23	6.81 × 10^-49^
MGM_05585	ATG13	Autophagy-related protein 13	295.8	9.10	1.43 × 10^-29^
MGM_06174	PGA13	GPI-anchored cell wall protein	11,682.1	5.17	1.57 × 10^-28^
MGM_00673	DIP5	Dicarboxylic amino acid permease	13,615.4	5.15	6.04 × 10^-26^
MGM_00695		Uncharacterized	110.6	10.57	2.59 × 10^-16^
MGM_04990		Uncharacterized	134.6	8.91	2.65 × 10^-11^
MGM_03596	HWP1	Hyphal cell wall protein	2223.7	5.38	2.2 × 10^-7^
MGM_03595	HWP1	Hyphal cell wall protein	6037.4	5.31	4.81 × 10^-7^
MGM_03825	FRE1	Ferric reductase transmembrane component	2044.7	6.87	1.81 × 10^-6^
MGM_03440	PHO89	Putative phosphate permease	1088.7	5.46	2.35 × 10^-4^
***CA3* vs. *CA2—downregulated genes***					
MGM_04452		Uncharacterized	15,450.6	−7.61	1.28 × 10^-127^
MGM_01056	FGR41	Putative GPI-anchored adhesin-like protein	7627.7	−8.12	3 × 10^-65^
MGM_01418	RBE1	Pry family cell wall protein	1370.4	−6.82	1.13 × 10^-52^
MGM_02468		Uncharacterized	1306.1	−5.06	5.69 × 10^-46^
MGM_04365	SCW11	Cell wall protein	2554.2	−5.08	6.42 × 10^-41^
MGM_01211		Uncharacterized	1072.9	−5.15	4.19 × 10^-32^
MGM_06317	CHI3	Chitinase	11,032.2	−8.77	2.24 × 10^-27^
MGM_04375		Uncharacterized	280.6	−5.57	1.75 × 10^-3^

^1^ Gene IDs are specific numbers derived from the *Candida albicans* reference genome P75063 (GCA_000775525) that can be found in the Ensembl Fungi database (https://fungi.ensembl.org/Candida_albicans_p75063_gca_000775525/Info/Index?db=core, accessed 24 October 2022). ^2,3^ Gene descriptions are based on the Candida Genome Database, but if information was not available there, the UniProt Database was used. The absence of gene names is caused by no hit in BLAST. Genes that are listed twice in the same category were separate transcripts annotated to the same gene by BLAST algorithm. ^4^ Mean of read counts in compared isolates.

**Table 2:** Differential expression characteristics of seven genes selected from comparisons regarding times of incubation in *Candida albicans* isolates (CA1, CA2, CA3).

Gene ID ^1^	Gene Name ^2^	Description ^3^	Mean Expression ^4^	Log2 (Fold Change)	*p*
***48 h*** **vs. *24 h—upregulated genes***					
MGM_04103	FRE1	Ferric reductase transmembrane component	1667.0	6.21	2.22 × 10^-6^
MGM_06015	ATX1	Putative cytosolic copper metallochaperone	10,752.6	2.20	3.13 × 10^-2^
MGM_03936	CSA2	Extracellular heme-binding protein	873.2	5.31	3.13 × 10^-2^
***72 h*** **vs. *24 h—upregulated genes***					
MGM_04103	FRE1	Ferric reductase transmembrane component	1667.0	8.00	4.11 × 10^-12^
MGM_03936	CSA2	Extracellular heme-binding protein	873.2	9.07	4.16 × 10^-10^
MGM_06015	ATX1	Putative cytosolic copper metallochaperone	10,752.6	2.78	6.7 × 10^-5^
MGM_05642		Uncharacterized	187.3	3.58	1.11 × 10^-4^
MGM_02771	GST1	Putative glutathione S-transferase 1	115.0	2.39	8.73 × 10^-3^
MGM_02719		Uncharacterized	108.2	2.96	9.69 × 10^-3^
MGM_00619	CSA1	Cell wall protein 1	11,289.7	3.79	9.69 × 10^-3^

^1^ Gene IDs are specific numbers derived from the *Candida albicans* reference genome P75063 (GCA_000775525) that can be found in the Ensembl Fungi database (https://fungi.ensembl.org/Candida_albicans_p75063_gca_000775525/Info/Index?db=core, accessed on 24 October 2022). ^2,3^ Gene descriptions are based on the Candida Genome Database, but if information were not available, on the UniProt Database. ^4^ Mean of read counts at compared time points.

## Data Availability

Data are available from the corresponding authors upon reasonable request.

## Supplementary Materials

The following supporting information can be downloaded at: https://www.mdpi.com/article/10.3390/ijms24010368/s1.
