# Supplementary material for: The Landscape of Gene Expression during Hyperfilamentous Biofilm Development in Oral Candida albicans Isolated from a Lung Cancer Patient
Source: Int J Mol Sci. 2022 Dec 26;24(1):368. doi: 10.3390/ijms24010368 (PMC9820384; doi:10.3390/ijms24010368)
Supplement: Supplementary file 1 [file ijms-24-00368-s001.zip › Figure S2.pdf]

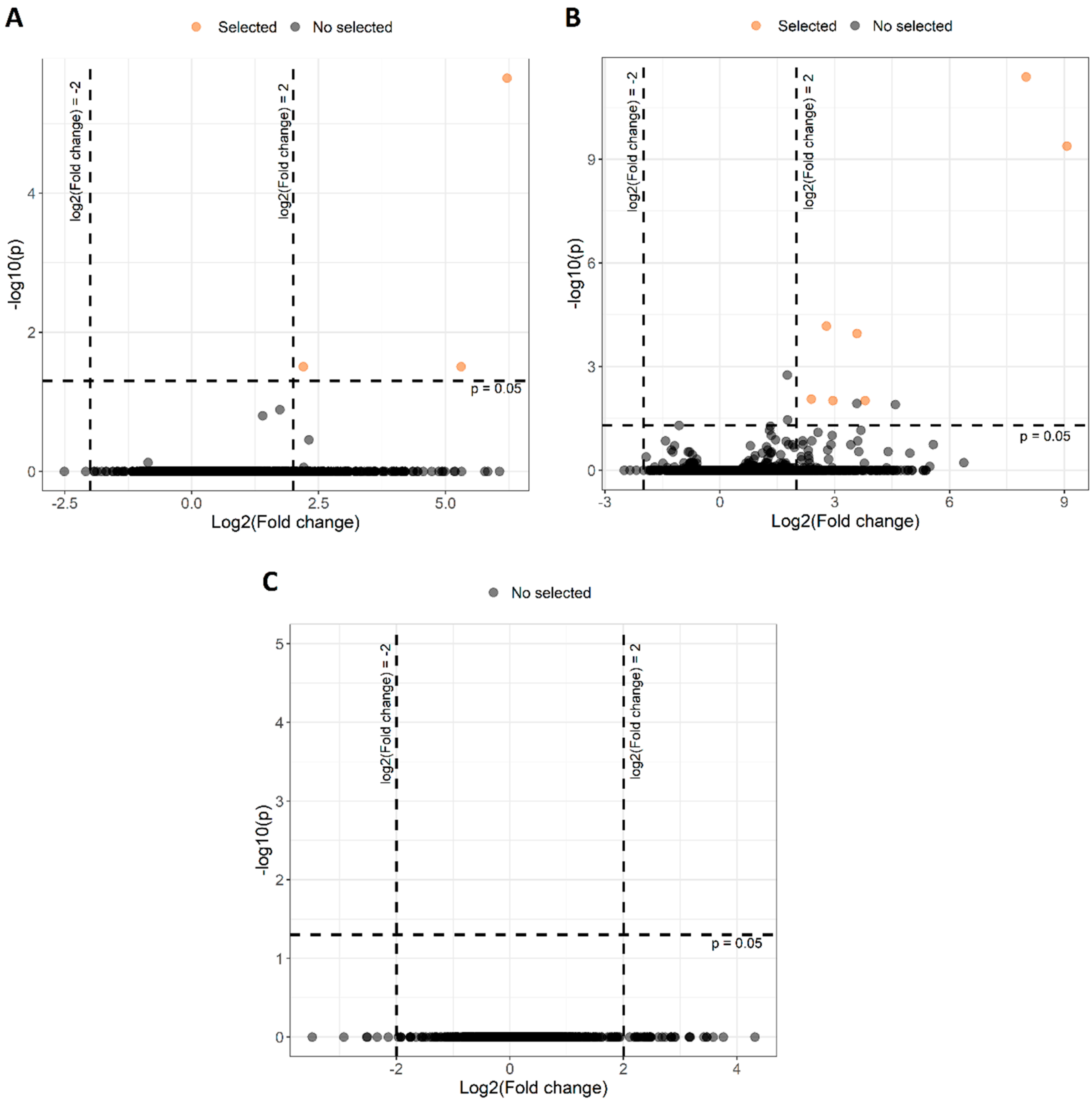

Figure S2. Volcano plots showing the arrangement of negative log10 of P values and log2 fold changes for 6,282 differentially expressed genes in (**A**) 24 h vs. 48 h comparison, (**B**) 72 h vs. 24 h comparison, and (**C**) 72 h vs. 48 h comparison. Genes marked as 'Selected' met three established selection criteria: mean of read counts above 100 across samples belonging to the compared groups, Benjamini-Hochberg adjusted P value below 0.05 and absolute log2(fold change) value above 2.
