# Supplementary material for: The Landscape of Gene Expression during Hyperfilamentous Biofilm Development in Oral Candida albicans Isolated from a Lung Cancer Patient
Source: Int J Mol Sci. 2022 Dec 26;24(1):368. doi: 10.3390/ijms24010368 (PMC9820384; doi:10.3390/ijms24010368)
Supplement: Supplementary file 1 [file ijms-24-00368-s001.zip › Figure S4.pdf]

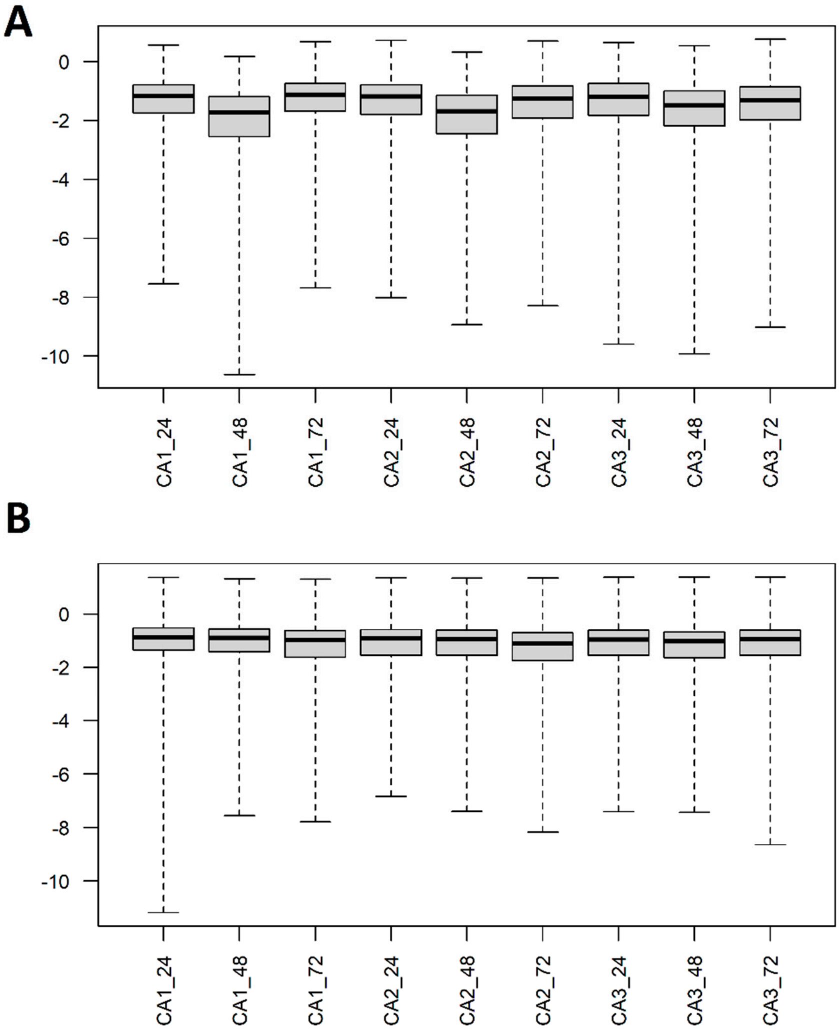

Figure S4. Boxplots of log10 Cook's distances of all genes analyzed in each sample using (**A**) isolates as a condition and (**B**) times of incubation as a condition. Whiskers define a range between minimum and maximum value of Cook's distance, boxes define a range between 25% and 75% quartile, and horizontal lines inside boxes indicate the median value. The first part of sample names indicates isolate (CA1, CA2, and CA3) and the second part of sample names indicates time of incubation (24 h, 48 h, and 72 h).
