# Supplementary material for: The Landscape of Gene Expression during Hyperfilamentous Biofilm Development in Oral Candida albicans Isolated from a Lung Cancer Patient
Source: Int J Mol Sci. 2022 Dec 26;24(1):368. doi: 10.3390/ijms24010368 (PMC9820384; doi:10.3390/ijms24010368)
Supplement: Supplementary file 1 [file ijms-24-00368-s001.zip › Figure S5.pdf]

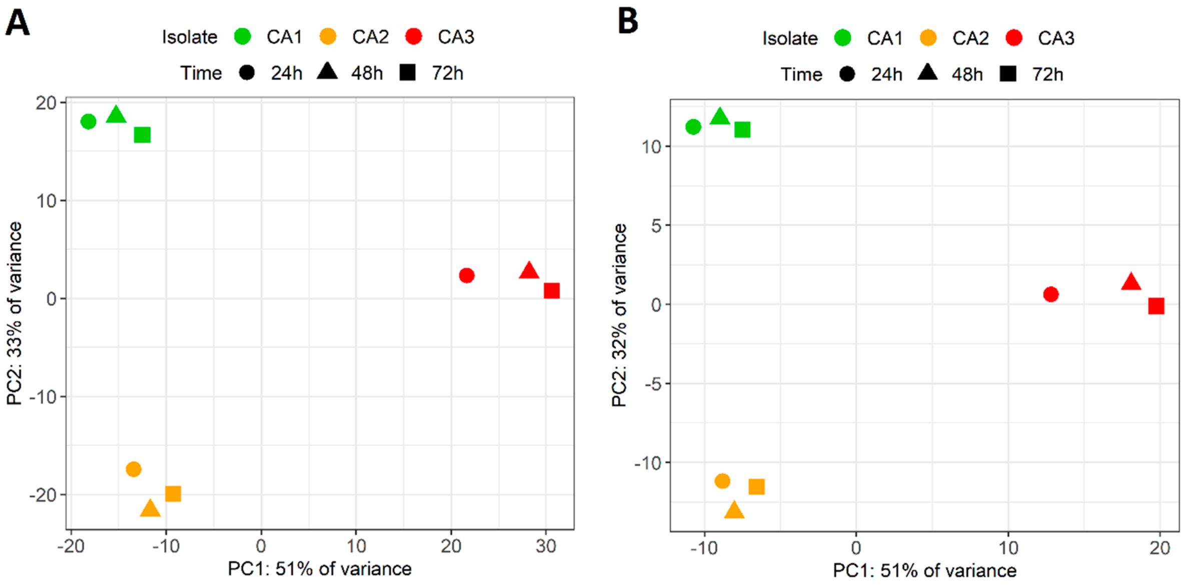

Figure S5. PCA plot of normalized and transformed (regularized log transformation) expression data of all genes analyzed in the studied samples using (**A**) isolates as a condition and (**B**) times of incubation as a condition.
