# Supplementary material for: The Landscape of Gene Expression during Hyperfilamentous Biofilm Development in Oral Candida albicans Isolated from a Lung Cancer Patient
Source: Int J Mol Sci. 2022 Dec 26;24(1):368. doi: 10.3390/ijms24010368 (PMC9820384; doi:10.3390/ijms24010368)
Supplement: Supplementary file 1 [file ijms-24-00368-s001.zip › Figure S6.pdf]

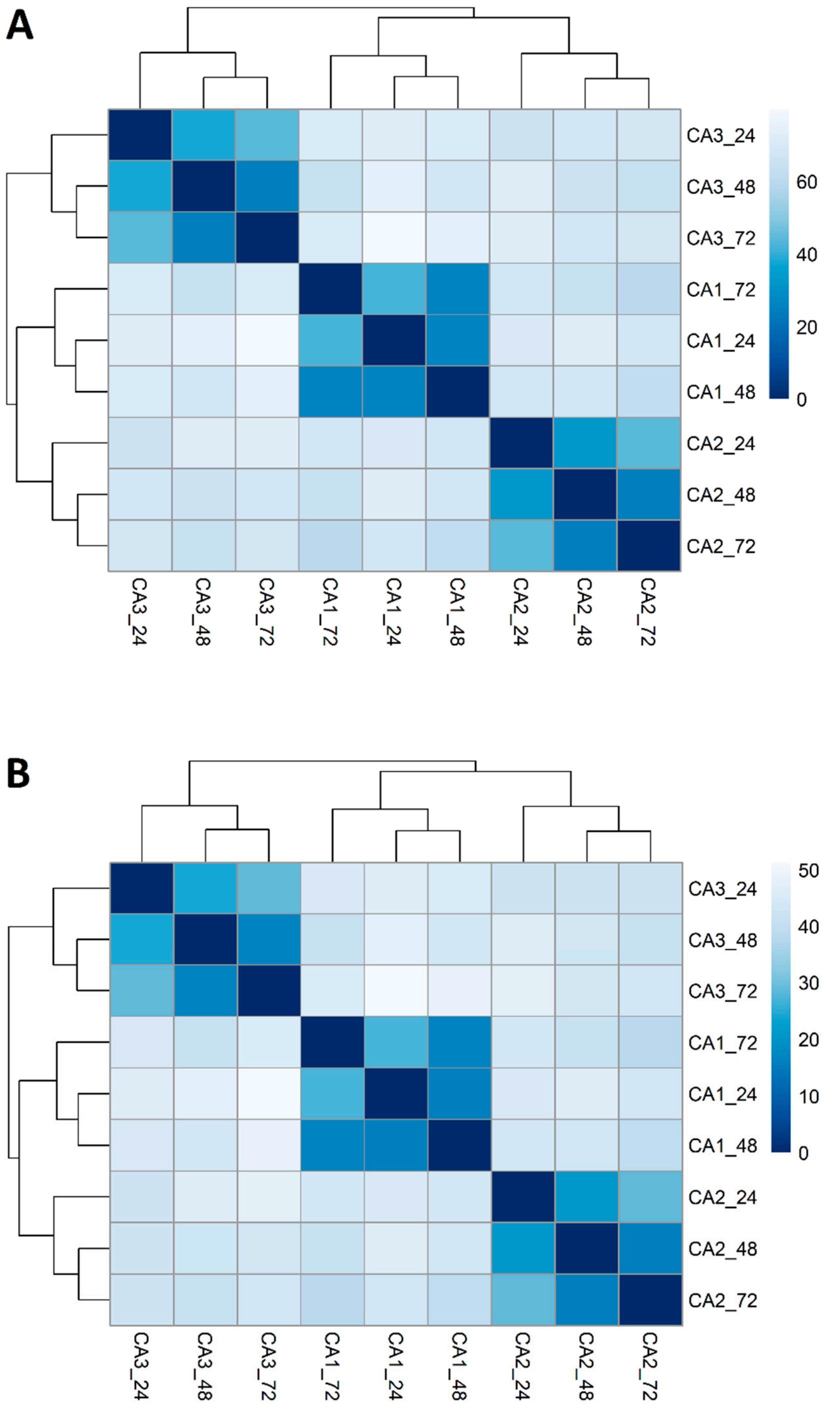

Figure S6. Heatmap of Euclidean distances clustering calculated for normalized and transformed (regularized log transformation) expression data of all genes analyzed in each samples using (**A**) isolates as a condition and (**B**) times of incubation as a condition. The first part of sample names indicates isolate (CA1, CA2, and CA3) and the second part of sample names indicates time of incubation (24 h, 48 h, and 72 h).
