# Supplementary figures and images for: The Landscape of Gene Expression during Hyperfilamentous Biofilm Development in Oral Candida albicans Isolated from a Lung Cancer Patient
Source: Int J Mol Sci. 2022 Dec 26;24(1):368. doi: 10.3390/ijms24010368 (PMC9820384; doi:10.3390/ijms24010368)

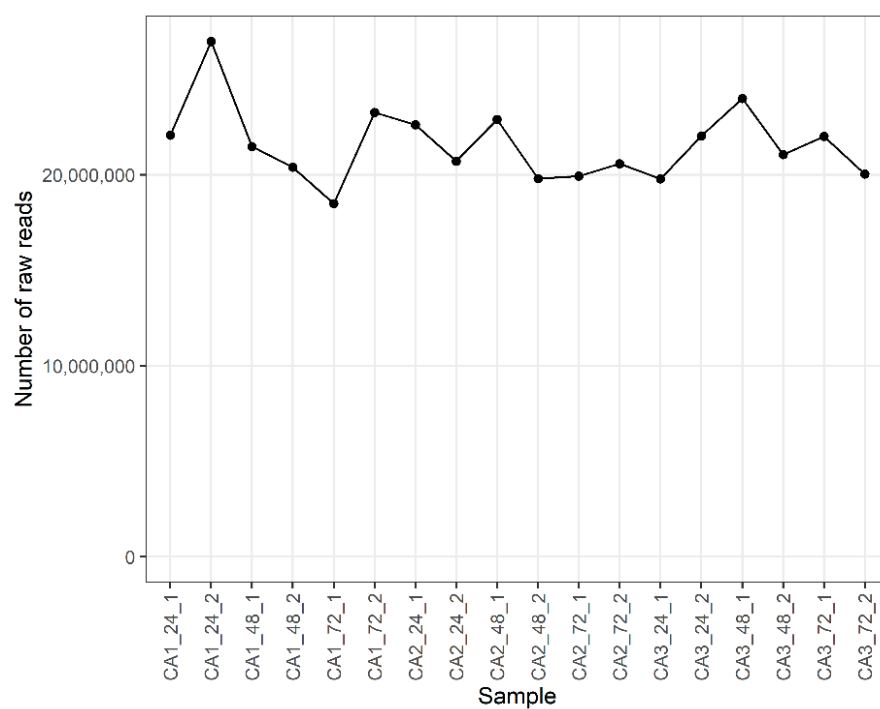

**Figure S3.** Numbers of raw reads obtained from the studied samples using RNA sequencing.

Supplement: Supplementary file 1 [file ijms-24-00368-s001.zip › Figure S3.pdf]
